# Correlation Between Indoxyl Sulfate in Chronic Kidney Disease and Olfactory Dysfunction 

**DOI:** 10.22038/ijorl.2024.77737.3632

**Published:** 2025

**Authors:** Irnanda-Warda-Rizki Nasution, Tengku-Siti-Hajar Haryuna, Delfitri Munir, Syafrizal Nasution, Putri-Chairani Eyanoer, Siti Nursiah, Ramlan Sitompul

**Affiliations:** 1 *Department of Otorhinolaryngology - Head and Neck Surgery, Faculty of Medicine, Universitas Sumatera Utara, Jl. Dr. Mansur No. 5, Medan, 20155, INDONESIA.*; 2 *Department of Internal Medicine, Faculty of Medicine, Universitas Sumatera Utara, Jl. Dr. Mansur No. 5, Medan 20155, INDONESIA.*; 3 *Department of Community Medicine, Faculty of Medicine, Universitas Sumatera Utara, Jl. Dr. Mansur No. 5, Medan, 20155, INDONESIA.*

**Keywords:** Chronic kidney disease, Indoxyl sulfate, Olfactory dysfunction, Sniffin’ stick test

## Abstract

**Introduction:**

Olfactory dysfunction is prevalent among individuals with chronic kidney disease (CKD), with prevalence escalating alongside disease severity. The uremic toxin we observed in this study is Indoxyl sulfate (IS), a potent uremic toxin that markedly accumulates in the plasma of patients with chronic insufficiency. Olfactory damage may occur in the setting of neuronal damage due to renal failure.

**Materials and Methods:**

27 patients, a total sample in this study with diagnosed chronic kidney disease within stage 5 on regular hemodialysis, were examined for indoxyl sulfate levels in blood plasma and then examined for their olfactory function using the Sniffin’ Sticks test. A correlation analysis was conducted between indoxyl sulfate levels and olfactory function test results in patients with CKD.

**Results:**

The Pearson correlation test revealed a strong, significant negative correlation between indoxyl sulfate levels and olfactory function (r = -0.613; p = 0.001). Additionally, correlations were found between indoxyl sulfate levels and each component of olfactory function: threshold value (r = -0.408; p = 0.035), discrimination (r = -0.807; p = 0.001), and identification (r = -0.703; p = 0.001).

**Conclusion:**

Olfactory function is compromised in individuals with chronic renal disease and correlates with the level of accumulation of the uremic toxin indoxyl sulfate.

## Introduction

Olfactory, the ability to feel and recognize smells, has a significant role in daily activities, especially the appreciation of taste, food intake, and recognition of danger (1). In prior studies, abnormalities in olfactory function have been documented in CKD patients, where these events affect function and well-being, thereby diminishing quality of life (1). Olfactory dysfunction is prevalent among individuals with CKD, and its prevalence escalates with the severity of the condition (2). It was found that 74% of patients with end-stage renal failure exhibited olfactory dysfunction (3). Another study indicated that 81% of individuals receiving renal replacement therapy who have end-stage renal failure experienced olfactory dysfunction (4). The study reported by Yusuf et al. showed that the majority of CKD patients with olfactory dysfunction have about 72% hyposmia and about 5% anosmia (2). Olfactory dysfunction pathophysiology in CKD patients is predominantly linked to uremia (5). Uremic toxins induce edema in the olfactory epithelial mucosa, diminishing sensitivity to odoriferous substances (5). Moreover, this toxin impairs the olfactory receptor cell regeneration, olfactory bulbs, and centers of the olfactory, leading to dysfunction of olfactory (5).

Indoxyl sulfate (IS) is a potent uremic toxin that significantly accumulates in the plasma of chronic kidney disease (CKD) patients, with concentrations potentially increasing up to 50-fold compared to healthy individuals, and it is not effectively eliminated by hemodialysis. It induces prooxidative and proinflammatory activity, activates a response of immunity, and promotes the progression of chronic kidney disease (CKD) (6). 

Research indicates a correlation between conversion in olfactory function and clinical indicators of malnutrition; however, no association was identified with the retention of uremic toxins, including monomethylamine, ethylamine, P-cresol sulfate, and indoxyl sulfate in chronic renal insufficiency patients undergoing hemodialysis (7). Research regarding the correlation of Indoxyl Sulfate levels to impaired olfactory function in patients with chronic kidney disease is still limited. This study analyzes the correlation of Indoxyl Sulfate value with diminished olfactory function in CKD patients. Based on the description above, we propose a hypothesis that olfactory dysfunction due to uremic syndrome, increased inflammatory markers, and oxidative stress that occurs in CKD have a relationship with Indoxyl Sulfate levels.

## Materials and Methods

This research is an observational analytic study employing a cross-sectional study design; this design aims to determine the correlation between plasma indoxyl sulfate levels and impaired olfactory function in patients with chronic kidney disease. Consecutive sampling was employed as a sampling technique, whereby all subjects who met the selection criteria were included in the study until the required sample size was achieved. 

This study was conducted in the hemodialysis room for examination of olfactory function using the sniffin’ stick test as well as in a fully equipped laboratory with sufficient expertise for ELISA examination of plasma indoxyl sulfate levels was carried out at the Integrated Laboratory Universitas Sumatera Utara, Medical Faculty.

*Sample*

The sample of this study were stage V CKD patients on regular hemodialysis in the Hemodialysis Room, Adam Malik General Hospital, where the diagnosis of CKD was made by an Internal Medicine Specialist Nephrology Consultant, aged <25 years - 55 years who did hemodialysis 2 times in 1 week, had kidney disease < 5 years to > 5 years with routine ENT examination results within normal limits. 

The exclusion criteria in this study were the patients with abnormal ENT physical examination such as infection, congestion, chronic rhinitis, asthma, upper respiratory infections, head trauma within the past two weeks, patients who were uncooperative during the examination, poor general condition and the patient who withdrew from the study. The minimum sample size is 27 patients, and the results were calculated using the proportion formula.

*Procedure*

To ensure the ethical integrity of all procedures in this study, the research proposal was submitted to the Research Ethics Commission Universitas Sumatera Utara for evaluation and approval, receiving ethical clearance under the number 1259/KEP/USU/2022.

The plasma levels of Indoxyl Sulfate (IS) were checked by taking a blood sample just before the patient underwent hemodialysis, as much as 3 mL, which was put into a tube containing EDTA. The supernatant portion is taken as a ready-to-test sample after centrifugation for 20 minutes at 3000 rpm. Plasma indoxyl sulfate levels can be measured immediately, or ready-to-test samples are stored in aliquots at -20°C or -80°C until IS levels are tested at the Integrated Laboratory. 

Then, the patients were examined for olfactory function using the Sniffin' Stick Test, which examined all components of the olfactory function. The "Sniffin' Sticks" (Burghart, Holm, Germany) was utilized for this study.

*Statistical Analysis*

The data distribution normality test was performed before the correlation test was performed using the Kolmogorov-Smirnov method. The Pearson correlation test is the appropriate correlation test for numerical data with normally distributed data (p>0.05). However, if the data is not normally distributed, a correlation test will be performed using Spearman's rho test. 

The correlation test will produce a correlation coefficient value as well as a significance value, which is stated to be significant if the p-value <0.05 using SPSS (Statistical Package for the Social Sciences).

## Results

This study investigated 27 subjects in total. The patients were in good condition and had routine hemodialysis with normal ENT examinations without a history of allergic rhinitis, CRS (Chronic Rhinosinusitis), asthma, or upper respiratory tract infection.

**Table 1:** Demographic Characteristics of Patients

**Demographic Characteristics**	**n = 27**
Sex, n (%)	
Male	**1** **7 (63%)**
Female	**10 (37%)**
**Age, year**	
**< 25 years-old**	**2 (7,4%)**
**26-35 years-old**	**4 (14,8%)**
**36-55 years-old**	**21 (77,8%)**
Duration of CKD	
< 5 years	**13 (48,1%)**
> 5 years	**14 (51,9%)**
**Urine Volume**	
** Normouria**	**6 (22,2%)**
** Oliguria**	**14 (51,9%)**
** Anuria**	**7 (25,9%)**

Based on Table 1, The characteristics of the patients were as follows. In sex group, most are male patients; 17 (63%), female patients; 10 (37%). The largest age group is between the ages of 36-55 years; 21 (77.8%), followed by 26-35 years age group; 4 (14,8%), and < 25 years age group; 2 (7,4%). Most of the patients suffered from CKD within a period of > 5 years; 14 (51.9%), < 5 years; 13 (48,1%). The majority of patients experienced decrease in urine volume with oliguria; 14 (51.9%), followed by anuria; 7 (25.9%), however, there were still subjects with normouria; 6 (22.2%).

**Table 2:** Olfactory function and Indoxyl sulfate value

**Olfactory function and Indoxyl sulfate value**	**n = 27**
Olfactory Function	
Mean (SD)	**23,17 (7,27)**
Median (Min-Max)	**22 (12 – 33)**
Normosmia	**8 (29,6%)**
Hyposmia	**11 (40,7%)**
**Anosmia** **Threshold** **Discrimination** **Identification**	**8 (29,6%)** 10 (37%) 18 (66%) 17 (62%)
**Indoxyl sulfate value**	
**Mean (SD)**	**78,16 (79,40)**
**Median (Min-Max)**	**35,12 (12,94 - 245,03)**

According to Table 2. The description of the olfactory function and indoxyl sulfate value in CKD patients. Most of the patients are hyposmia; 11 (40.7%), followed by normosmia; 8 (29.6%) and anosmia; 8 (29.6%), with olfactory threshold; 10 (37%), discrimination; 18 (66%), and identification; 17 (62%) out of 27 patients experienced decrease in function. We found high plasma levels of indoxyl sulfate with mean plasma level of indoxyl sulfate at 78.16 g/ml, a standard deviation of 79.40 g/ml, plasma level of indoxyl sulfate with the lowest value of 12.94 g/ml, and the highest value of 245.03 g/ml.

**Table 3:** Correlation between Indoxyl Sulfate with Olfactory Function

**Correlation between Indoxyl Sulfate with Olfactory Function**	**n = 27**	**n = 27**
	r value	p value
Olfactory Function (TDI)	**-0,613**	**0,001**
Threshold	**-0,408**	**0,035**
**Discrimination**	**-0,807**	**0,001**
**Identification**	**-0,703**	**0,001**

In Table 3 showed a weak significant correlation on indoxyl sulfate levels with a threshold value (r= -0.408; p=0.035). However, there are robust and strong significant correlation on discrimination value (r= -0.807; p = 0.001) and identification (r= -0.703; p=0.001) of olfactory function. There is also a significant correlation indicated between indoxyl sulfate levels and olfactory function as the total amount of olfactory threshold (T), discrimination (D), and identification (I) (r= -0.613; p=0.001). In other words, there is a correlation between level of indoxyl sulfate and olfactory dysfunction in CKD patients.

## Discussion

Olfactory dysfunction pathophysiology in chronic renal insufficiency patients is predominantly linked to uremia. Uremic toxins induce edema in the olfactory epithelial mucosa, consequently diminishing sensitivity to odors (5). Moreover, this toxin impairs the regeneration of olfactory receptor cells, olfactory bulbs, and olfactory centers, leading to olfactory dysfunction (5). IS is the terminal product of the dietary metabolism of tryptophan, due to its strong albumin-binding affinity, is not effectively eliminated by hemodialysis. Additionally, it exhibits prooxidative and proinflammatory properties, incites a response immunity, and promotes the progression of CKD (6).

This study found that most of the patients were male (63%), in the 36-55 age group (77.8%). Most of the patients suffered from CKD within a period of > 5 years (51.9%). The 11th Indonesian Kidney Registry Report states that the prevalence of CKD in men in Indonesia is greater than that of women and states that the highest proportion of patients are still in the 45-64-year age category (8). Patients under the age of 25 accounted for 2.57% of active patients (8), this shows that it is time to pay attention to the younger age group to start paying attention to kidney health. Most samples experienced a decrease in urine volume with oliguria (51.9%). This study did not find any significant relationship between the duration of CKD (r= -0.127; p =0.250) and urine volume (r= 0.196; p =0.137) with olfactory disorders but found a significant relationship between the duration of CKD (r= 0.494; p =0.009) and urine volume (r= -0.424; p =0.028) with the value of indoxyl sulfate. This supports the theory that has been put forward as stated by Vanholder et al., uremic toxin is a chemical that is usually filtered by healthy kidneys, but after a decrease in kidney function, the toxin multiplies in the body and interacts negatively with the body's biological functions (9).

Research conducted by Koseoglu et al. in 83 CKD patients found 55 patients with hyposmia (74.6%), seven patients (8.43%) experienced anosmia, and 14 patients with normosmia (16.8%), states that there is a disturbance of olfactory function in patients with CKD (10). According to the results of this study, it was found that the majority of CKD sufferers experienced hyposmia, 11 patients (40.7%). Subjects with anosmia, 8 patients (29.6%), and subjects with normal olfactory function, 8 patients (29.6%).

This study obtained the relationship between indoxyl sulfate values and each component of the olfactory function. The result was that a weak, significant negative correlation was found between the indoxyl sulfate value and the threshold value (r = -0.408; p = 0.035). At the same time, the results of measurements of indoxyl sulfate values with discrimination and identification of the olfactory function showed that there was a robust and strong significant negative correlation between indoxyl sulfate values, discrimination (r= -0.807; p =0.001) and identification (r= -0.703; p = 0.001). The results of measuring the overall threshold value, discrimination, and identification (ADI) of olfactory function with Indoxyl Sulfate levels using the Pearson correlation test showed that there was a significant negative correlation between indoxyl sulfate values and olfactory function (r = -0.613; p = 0.001).

A study by Griep et al. revealed a significant positive association between olfactory function and creatinine clearance in patients with different degrees of kidney insufficiency and transplant recipients (p = 0.02) (4). A notable negative correlation was identified between olfactory function and creatinine clearance (p = 0.02), olfactory function and serum urea concentrations (p < 0.001), protein catabolic rate (p < 0.05), and serum phosphorus (p = 0.022 ) (4). 

Additional parameters assessing nutritional status (BMI, albumin) exhibited no correlation with olfactory function (4). This study's results demonstrate that olfactory function is significantly compromised in patients with this disease, correlating with the extent of renal damage and the accumulation of uremic toxins (4). Following renal transplantation, the patient exhibits normal olfactory function, suggesting that the olfactory system can recover when uremic toxin levels fall below a critical threshold. However, the acute removal of uremic toxins through dialysis does not ameliorate olfactory impairments, as evidenced by the persistent uremia impact on olfactory function. Otherwise, a study conducted by Raff indicated that there was no significant correlation between impaired olfactory function and malnutrition in chronic kidney disease patients concerning four examined uremic toxins: indoxyl sulfate, ethylamine, monomethylamine, and P-cresol sulfate (7). Their findings indicate a correlation between inadequate nutritional status and diminished olfactory function in patients with severe kidney insufficiency. Further investigation is required to identify the uremic toxins that facilitate these processes (7). Indoxyl sulfate exhibits significant protein binding, resulting in a markedly low hemodialytic clearance compared to urea, as only free unbound solutes can permeate the dialyzer membrane (11). In standard treatment, the clearance of indoxyl sulfate ranges from 25 to 30 mL/min, while the clearance of urea exceeds 200 mL/min (11). Therefore, plasma indoxyl sulfate levels increase to levels higher than those of urea in hemodialysis patients (11).

**Fig 1:**
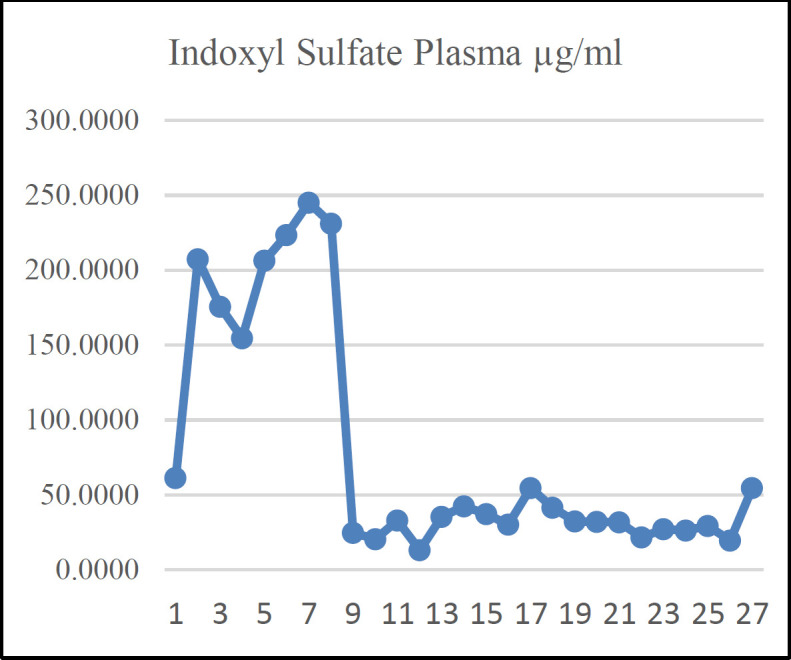
Concentration of Indoxyl Sulfate Plasma ( g/ml) in CKD patients

This study showed that patients with CKD had high plasma levels of indoxyl sulfate with an average plasma level of indoxyl sulfate 78.16  g/ml with a standard deviation of 79.40  g/ml, plasma levels of indoxyl sulfate with the lowest value of 12.94  g/ml and the highest 245.03  g/ml showed in Figure 1. Indoxyl sulfate is the level of indoxyl sulfate in the blood, both plasma and serum. Until now, there has been no international consensus on the normal value of Indoxyl Sulfate (IS) levels. However, in several studies examining Indoxyl Sulfate (IS) levels in healthy subjects, the levels were close to zero (12). Free indoxyl sulfate concentrations in predialysis patients can be 75-116 times higher than those in healthy subjects (13).

Gao and Liu stated that indoxyl sulfate has been recognized as capable of stimulating oxidative stress, which will ultimately contribute to the development of vascular disorders in CKD patients. Increased oxidative stress is related to an imbalance between ROS production and degradation (13). Edamatsu et al. also stated that oxidative stress is triggered by increased pro-oxidants and decreased antioxidants (14). Indoxyl sulfate induces the production of ROS (Reactive Oxygen Species) in various cells by activating NADPH oxidase and decreasing glutathione levels (15).

**Fig 2:**
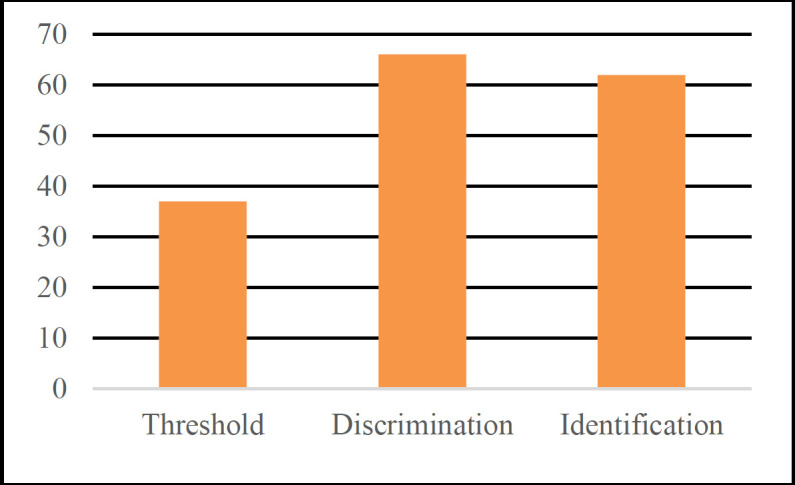
Percentage of patients. Individual tests of olfactory function were affected to different degrees.

In this study, it was shown that in CKD patients were found a significant olfactory disturbance of 70.3%, which occurred without the patient realizing it himself. It is possible that CKD patients will suffer from impaired olfactory function with various scales, as can be seen in Figure 2. Until now, researchers have not found studies specifically looking for a relationship or correlation between indoxyl sulfate levels and olfactory function. Decreased sensitivity of olfactory function in patients with chronic renal failure is often not realized by the patients; in the end, olfactory function will decrease and is a characteristic of the disease. In this study, olfactory disturbances were found in patients with chronic kidney disease limited to the function of discrimination and identification of smell, but the threshold function was not as severe as the other two components. Thus, this pattern reflects that the processing of discrimination and identification of smell is in the central pathway, while the processing of the olfactory threshold reflects the peripheral olfactory pathway. The same pattern was also found in a study by Frasnelli et al., which found that olfactory impairment in hemodialysis patients had markedly impaired discrimination and identification values but not the olfactory threshold value (16).These findings led to speculation that the impaired olfactory function in patients with chronic renal failure originates at the central nervous level (14). Accumulation of a few toxins, such as uric acid, p-cresol sulfate, IL-6, IL-1, indoxyl sulfate, parathormone, and TNF-alpha, has a negative effect on the central nervous system (17). The accumulated metabolic substances cannot be cleared from the body through peritoneal dialysis or hemodialysis. Thus, small urea compounds remain in the blood when the patient has undergone hemodialysis (10).

## Conclusion

We conclude that there is a significant negative correlation between indoxyl sulfate levels and impaired olfactory function. This supports the statement that olfactory ability is impaired in patients with chronic kidney disease and is associated with the level of indoxyl sulfate accumulation as a uremic toxin. Furthermore, further research is needed to conduct interventions in terms of whether or not it can reduce the value of indoxyl sulfate, which can interfere with olfactory function.
